# Increase of serum uric acid levels associated with *APOE* ε2 haplotype: a clinico-genetic investigation and in vivo approach

**DOI:** 10.1007/s13577-021-00609-w

**Published:** 2021-09-16

**Authors:** Masatsune Ogura, Yu Toyoda, Masayuki Sakiyama, Yusuke Kawamura, Akiyoshi Nakayama, Yoshihide Yamanashi, Tappei Takada, Seiko Shimizu, Toshihide Higashino, Mayuko Nakajima, Mariko Naito, Asahi Hishida, Sayo Kawai, Rieko Okada, Makoto Sasaki, Makoto Ayaori, Hiroshi Suzuki, Koki Takata, Katsunori Ikewaki, Mariko Harada-Shiba, Nariyoshi Shinomiya, Hirotaka Matsuo

**Affiliations:** 1 Department of Metabolism and Endocrinology, Eastern Chiba Medical Center, 3-6-2 Okayamadai, Togane, Chiba 283-8686 Japan; 2 Department of General Medical Science, Chiba University Graduate School of Medicine, 1-8-1 Inohana, Chuo-ku, Chiba, Chiba 260-8670 Japan; 3 Department of Molecular Innovation in Lipidology, National Cerebral and Cardiovascular Center Research Institute, 6-1 Kishibe-shinmachi, Suita, Osaka 564-8565 Japan; 4 Department of Integrative Physiology and Bio-Nano Medicine, National Defense Medical College, 3-2 Namiki, Tokorozawa, Saitama 359-8513 Japan; 5 Department of Dermatology, National Defense Medical College, 3-2 Namiki, Tokorozawa, Saitama 359-8513 Japan; 6 Department of Pharmacy, Faculty of Medicine, The University of Tokyo Hospital, The University of Tokyo, 7-3-1 Hongo, Bunkyo-ku, Tokyo, 113-8655 Japan; 7 Department of Preventive Medicine, Nagoya University Graduate School of Medicine, 65 Tsurumai-cho, Showa-ku, Nagoya, Aichi 466-8550 Japan; 8 Department of Oral Epidemiology, Hiroshima University Graduate School of Biomedical and Health Sciences, 1-2-3 Kasumi, Minami-ku, Hiroshima, 734-8553 Japan; 9 Department of Public Health, Aichi Medical University School of Medicine, 1-1 Yazako-karimata, Nagakute, Aichi 480-1195 Japan; 10 Division of Anti-Aging and Vascular Medicine, Department of Internal Medicine, National Defense Medical College, 3-2 Namiki, Tokorozawa, Saitama 359-8513 Japan; 11 Takata Clinic, 10-15 Wakakusa-cho, Higashi-ku, Hiroshima, Hiroshima 732-0053 Japan

**Keywords:** Apolipoprotein, Human study, Menopause status, Single-nucleotide polymorphisms (SNPs), Urate

## Abstract

Elevated serum uric acid (SUA)—hyperuricemia—is caused by overproduction of urate or by its decreased renal and/or intestinal excretion. This disease, which is increasing in prevalence worldwide, is associated with both gout and metabolic diseases. Several studies have reported relationships between *apolipoprotein E* (*APOE*) haplotypes and SUA levels in humans; however, their results remain inconsistent. This prompted us to investigate the relationship between *APOE* polymorphisms and SUA levels. Our subjects were 5,272 Japanese men, premenopausal women, and postmenopausal women. Multiple linear regression analyses revealed the ε2 haplotype of *APOE* to be independently associated with higher SUA in men (*N* = 1,726) and postmenopausal women (*N* = 1,753), but not in premenopausal women (*N* = 1,793). In contrast, the ε4 haplotype was little related to SUA levels in each group. Moreover, to examine the effect of Apoe deficiency on SUA levels, we conducted animal experiments using *Apoe* knockout mice, which mimics ε2/ε2 carriers. We found that SUA levels in *Apoe* knockout mice were significantly higher than those in wild-type mice, which is consistent with the SUA-raising effect of the ε2 haplotype observed in our clinico-genetic analyses. Further analyses suggested that renal rather than intestinal underexcretion of urate could be involved in Apoe deficiency-related SUA increase. In conclusion, we successfully demonstrated that the ε2 haplotype, but not the ε4 haplotype, increases SUA levels. These findings will improve our understanding of genetic factors affecting SUA levels.

## Introduction

Hyperuricemia is caused by the overproduction of urate or by decreased renal [1, 2] and intestinal [3, 4] urate excretion. This common disease is not only associated with gout but also with other common conditions including hypertension [5, 6] and atherosclerotic cardiovascular diseases [7] as well as kidney diseases [8]. Although recent studies have revealed the pathophysiological importance of urate transporters in urate handling in humans [9, 10], other (non-transporter) genetic factors associated with serum uric acid (SUA) levels have also been reported [11–13]. Moreover, based on the classically known association between hyperuricemia and hyperlipidemia [14, 15], the influence on SUA levels of genetic factors affecting lipid levels in the blood is likely to be involved. One of these is variation in *apolipoprotein E* (*APOE*) polymorphisms; however, as described below, their effects on SUA levels have not been conclusive.

The human *APOE* gene, which is located on chromosome 19q13.2, has two common non-synonymous single-nucleotide polymorphisms (SNPs)—rs429358 (c.334T>C; p.Cys112Arg) and rs7412 (c.472C>T; Arg158Cys) [16]. Given the lack of simultaneous presence of their minor alleles in one haplotype, three haplotypes are defined, named as ε2, ε3, and ε4. Six diplotypes have been observed as combinations of these three haplotypes (Table 1). Among the three haplotypes, ε3 is the commonest and recognized as the parent form, corresponding to the wild type (WT).

**Table 1:** Haplotypes of two common variants of human *APOE* gene

Haplotypes	rs429358 (Cys112Arg)	rs7412 (Arg158Cys)
ε2	T (Cys)	T (Cys)
ε3	T (Cys)	C (Arg)
ε4	C (Arg)	C (Arg)

APOE, a glycoprotein constituted of 299 amino acids, is chiefly distributed in very low-density lipoproteins (VLDLs), chylomicrons, and some high-density lipoproteins (HDLs) [17]. It plays multiple roles in the regulation of lipid and lipoprotein levels in the blood [18]. *APOE* polymorphisms are also reportedly associated not only with lipoprotein metabolism but with atherosclerotic cardiovascular diseases [19], kidney diseases [20], and neurodegenerative diseases [21–24]. Accordingly, given the observed associations between SUA levels and these disease phenotypes, it is possible that *APOE* polymorphisms and SUA levels are confounding factors for these disorders. Investigation of the latent relationship between *APOE* polymorphisms and SUA levels in humans should therefore provide new insights into the pathogenesis of hyperuricemia as well as its associated diseases. Several studies have investigated the relationship between *APOE* polymorphisms and SUA levels, but their results remain inconsistent.

Hitherto, several human studies have reported that the ε2 and ε4 haplotypes may be associated with higher [25–28] and lower [29] SUA levels, respectively, than seen with the ε3 haplotype. In contrast, other studies have reported an association between the ε4 haplotype and higher SUA levels [30, 31]. To enhance our understanding of this unresolved question, we herein aimed to investigate the relationships between *APOE* polymorphisms and SUA levels in a larger population. We also performed animal experiments using *Apoe* knockout (KO) mice to examine the effect of total Apoe deficiency on SUA levels in terms of urate excretion from the body.

## Methods

### Study participants

All the procedures used in human studies were approved by the institutional ethical committees (National Defense Medical College and Nagoya University), and were performed in accordance with the Declaration of Helsinki. All of the Japanese individuals in this study were recruited from participants in the Shizuoka area and Daiko area in the Japan Multi-Institutional Collaborative Cohort Study (J-MICC Study) [32, 33]. Written informed consent was obtained from all the subjects.

Among the participants, those who were under treatment for or had past histories of gout/hyperuricemia or dyslipidemia, and female participants for whom there was no information about menopause were excluded. Multiple regression analysis was performed on the resulting 5,272 subjects to evaluate the relationships among SUA levels, *APOE* gene polymorphisms, and other risk factors. Non-HDL cholesterol level was calculated using the following equation: [Non-HDL cholesterol (non-HDL-C) (mg/dL)] = [Total cholesterol (TC) (mg/dL)] − [HDL cholesterol (HDL-C) (mg/dL)].

### Genetic analysis

Genomic DNA was extracted from whole peripheral blood cells. Genotyping of the two SNPs (rs429358 and rs7412) in the *APOE* gene was performed using the TaqMan method (Thermo Fisher Scientific, Waltham, MA, USA) with a LightCycler 480 (Roche Diagnostics, Mannheim, Germany) [34], with minor modifications. To confirm the genotypes, direct sequencing was also performed on more than 200 samples with the following primers: for forward, 5′-CCTACAAATCGGAACTGGAG-3′, and for reverse, 5′-CCCGGCCTGGTACACTG-3′. DNA sequencing analysis was performed with a 3130xl Genetic Analyzer (Thermo Fisher Scientific) [34].

### Experimental assessment of urate excretion pathways

Animals were handled humanely in accordance with the National Cerebral and Cardiovascular Center’s Guidelines for the Care and Use of Laboratory Animals. The experimental protocol and animal use procedures were approved by the Committee of the National Cerebral and Cardiovascular Center. Male *Apoe* KO mice [35] (Jackson Laboratories, Bar Harbor, ME, USA) and control WT mice (C57BL6/J; Japan SLC, Shizuoka, Japan) were fed a normal rodent laboratory diet (CE-2; CLEA Japan, Tokyo, Japan).

Concentrations of urate and creatinine in collected serum and urine samples were determined by QuantiChrom Uric Acid Assay Kit (BioAssay Systems, Hayward, CA, USA) and Creatinine Assay Kit (Cayman Chemical, Ann Arbor, MI, USA), respectively. To analyze intestinal urate excretion, mice that had fasted overnight were anaesthetized by intraperitoneal injection of urethane and cannulated with polyethylene tubing (Hibiki Size 8) (Sansyo, Tokyo, Japan) at the upper duodenum and the middle jejunum to make an intestinal loop at the upper half of the intestine, in the same way as in our previous study [4]. After the intestinal contents had been removed by the slow infusion of saline and air, efflux buffer (saline containing 0.3 mM potassium oxonate) was introduced into the intestinal loop and both ends of the loop were closed with syringes. After the indicated periods, the efflux buffer in the loop was collected using syringes and the urate concentrations were quantified. Intestinal urate excretion was calculated using the following equation: [Intestinal urate excretion] = [Urate concentration in the intestinal loop] × [Volume of efflux buffer in the intestinal loop] × [Length of the whole small intestine]/[Length of the intestinal loop] as previously described [4].

### Statistical analysis

For statistical analysis calculations in the human studies, R software (version 3.1.1) (http://www.r-project.org/) was used. Student’s *t* test was employed for comparison of SUA levels in humans. We also carried out a multiple regression analysis to evaluate the independent effect of *APOE* polymorphisms on SUA levels, adjusting for confounding factors such as serum creatinine levels and non-HDL cholesterol levels. In animal experiments, analyses were performed using JMP software version 12.0 (SAS Institute, Cary, NC, USA). Student’s *t* test was used for comparison of urate concentrations in mice. All *P* values were two-tailed, and a *P* value of < 0.05 was considered to be statistically significant.

## Results

### Effects of *APOE* haplotypes on SUA levels in humans

To investigate the effects of *APOE* haplotypes on SUA levels, we examined the associations between *APOE* haplotypes and SUA levels in 5,272 Japanese individuals. SUA levels in carriers and non-carriers of *APOE* ε2 and ε4, respectively, are summarized in Tables 2 and 3. The call rates for two SNPs (rs429358 and rs7412) that determine the *APOE* haplotypes were 100%; these SNPs in the control group were in Hardy–Weinberg equilibrium (*P* > 0.05). Due to sex differences in SUA levels, which are also affected by menopause, we divided the study participants into three groups: men (*N* = 1,726), premenopausal women (*N* = 1,793), and postmenopausal women (*N* = 1,753). As shown in Table 2, SUA levels were significantly higher in postmenopausal than in premenopausal women (*P* = 3.1 × 10^−52^). The ε2 haplotype was associated with higher SUA levels in men (*P* = 0.033) and in postmenopausal women (*P* = 0.048); however, interestingly, the ε2 haplotype did not affect SUA levels among premenopausal women (*P* = 0.61) (Table 2). The ε4 haplotype was not significantly related to SUA levels in men, premenopausal women, or postmenopausal women (Table 3). We therefore conducted further analyses focusing on the ε2 haplotype.

**Table 2:** SUA levels of *APOE* ε2 carriers and non-ε2 carriers among 5,272 individuals

	Men			Premenopausal women			Postmenopausal women		
	*N*	SUA	*P* value	*N*	SUA	*P* value	*N*	SUA	*P* value
ε2 carrier	142	6.27 ± 1.22		168	4.05 ± 0.91		171	4.68 ± 0.97	
Non-ε2 carrier	1,584	6.04 ± 1.24	0.033	1,625	4.08 ± 0.83	0.61	1,582	4.53 ± 0.96	0.048
Total	1,726	6.06 ± 1.24		1,793	4.08 ± 0.84		1,753	4.55 ± 0.96	

Data are expressed as mean ± SD

*SUA* serum uric acid

**Table 3:** SUA levels of *APOE* ε4 carriers and non-ε4 carriers among 5,272 individuals

	Men			Premenopausal women			Postmenopausal women		
	*N*	SUA	*P* value	*N*	SUA	*P* value	*N*	SUA	*P* value
ε4 carrier	317	6.05 ± 1.15		370	4.09 ± 0.83		285	4.51 ± 0.91	
Non-ε4 carrier	1,409	6.06 ± 1.26	0.89	1,423	4.07 ± 0.84	0.77	1,468	4.55 ± 0.97	0.45
Total	1,726	6.06 ± 1.24		1,793	4.08 ± 0.84		1,753	4.55 ± 0.96	

Data are expressed as mean ± SD

*SUA* serum uric acid

To examine the quantitative effect on SUA levels of harboring the ε2 haplotype, we next performed a multiple linear regression analysis that included variables associated with increased SUA levels. A previous study showed that in healthy adults, the correlation coefficient for an association of non-HDL cholesterol with SUA was higher than those for associations of triglycerides and other lipid parameters including TC, HDL-C, and LDL-C [36]. Based on this information, we chose non-HDL-C levels as a covariate among available lipid parameters. In the multiple linear regression analysis adjusted for age, body mass index (BMI), serum creatinine levels, and non-HDL-C levels, the ε2 haplotype was independently associated with higher SUA levels in men (*P* = 0.015) and postmenopausal women (*P* = 0.005), while this was not in the case in premenopausal women (*P* = 0.55) (Table 4). Although these associative analyses could not uncover the molecular mechanisms lying behind the *APOE* ε2-associated increase of SUA levels, the fact that the APOE E2 protein is defective in its binding ability to the APOE receptors, unlike APOE E4 protein [37] and APOE E3 protein [38], suggests that APOE dysfunction might lead to increased SUA. To address this hypothesis, we further conducted in vivo analyses using male *Apoe* KO mice as described below.

**Table 4:** Effect of *APOE* ε2 haplotype and other risk factors on SUA levels in 5,272 individuals

	Men			Premenopausal women			Postmenopausal women		
	*β*	SE	*P* value	*β*	SE	*P* value	*β*	SE	*P* value
ε2 haplotype of *APOE*	0.25	0.10	0.015	− 0.039	0.065	0.55	0.21	0.070	0.0050
Age	− 0.0090	0.0030	0.0020	− 0.0020	0.0037	0.58	0.0020	0.0040	0.60
BMI	0.070	0.010	3.4 × 10^−12^	0.070	0.0060	3.5 × 10^−28^	− 0.000012	0.000021	0.59
Serum creatinine	2.59	0.23	1.7 × 10^−29^	2.38	0.22	2.2 × 10^−26^	2.81	0.24	1.2 × 10^−30^
Non-HDL-C	0.0041	0.00080	1.7 × 10^−6^	0.0022	0.00070	0.001	0.0045	0.00070	4.6 × 10^−11^

*BMI* body mass index, *non-HDL-C* non high-density lipoprotein cholesterol, *SE* standard error

### Effects of *Apoe* knockout on SUA levels and urate secretion in mice

To examine whether deficiency in Apoe function can affect SUA levels, we performed animal experiments using male *Apoe* KO mice. Given that like APOE deficiency, ε2 homozygosity is also associated with an increased risk of type III hyperlipoproteinemia in humans [36], together with the fact that knock-in mice carrying the human APOE E2 allele in place of the mouse *Apoe* gene cause type III hyperlipoproteinemia and spontaneous atherosclerosis in mice [39], *Apoe* KO mice can be a model mimicking ε2/ε2 carriers.

**Fig. 1:**
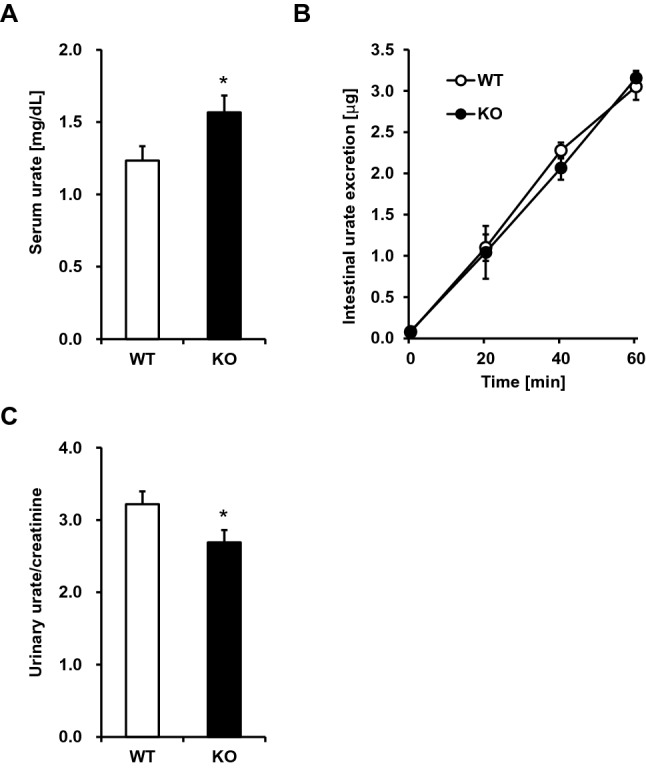
Serum uric acid levels and urate excretion activities in *Apoe* knockout mice. **A** Serum uric acid levels. **B** Intestinal urate excretion. There were no significant differences in intestinal urate excretion between WT and KO mice at each time point. **C** Renal urate excretion. *WT* wild type mice, *KO* *Apoe* knockout mice. Data are expressed as mean ± standard error (SE). *n* = 12 (**A**), 9 (**B**), and 12 (**C**). *P < 0.05 (*t* test)

As expected, SUA levels of *Apoe* KO mice were significantly higher than those of WT mice (*P* = 0.021) (Fig. 1A). We then investigated the latent mechanisms in terms of urate excretion from the body. As shown in Fig. 1B, there was no significant difference in urate excretion from the intestine between *Apoe* KO mice and WT mice; however, the urinary urate/creatinine ratios were significantly lower in *Apoe* KO mice than those in WT mice (*P* = 0.022) (Fig. 1C). There was no difference in body weight between *Apoe* KO mice and WT mice (28.1 ± 1.0 g vs*.* 28.0 ± 3.2 g; *P* = 0.937). These results suggest that higher SUA levels in *Apoe* KO mice could be caused by a decrease not in intestinal but in renal urate excretion.

## Discussion

The present study demonstrates the ε2 haplotype, not ε4 haplotype, to be associated with higher SUA levels in a Japanese population (Tables 2 and 3). This association was observed in male subjects and in postmenopausal female subjects, but not in premenopausal female subjects. To the best of our knowledge, this is the first report showing the effect of the ε2 haplotype on SUA levels to be different in women between before and after menopause. In other words, in contrast to the ε4 haplotype, the ε2 haplotype can have different effects on human SUA levels in difference populations, possibly in response to their levels of sex hormones. Although this study cannot support a previously reported association between the ε4 haplotype and lower SUA levels in southern Iranian subjects [29], our results are consistent with several previous studies showing that the ε2 haplotype was associated with higher SUA levels in Caucasian [20, 28] and Chinese [25, 26] populations.

Our findings suggest that the presence of a menstrual cycle, absent in men and postmenopausal women, might attenuate the effect of the ε2 haplotype causing higher SUA levels in premenopausal women. In women, the mean values of SUA levels reportedly increase after menopause [40], suggesting that female hormone-mediated regulatory mechanisms are involved in lowering SUA. This notion is supported by a mechanistic insight [41] showing that in the kidney of mice, estradiol and progesterone suppressed the protein levels of physiologically important renal urate re-absorbers [i.e., urate transporter 1 (Urat1/Slc22a12) [42] and glucose transporter 9 (Glut9/Slc2a9) [43]] and sodium-coupled monocarboxylate transporter 1 (Smct1/Slc5a8, a functional co-operator of Urat1 [44]), respectively, which could theoretically result in an increase in renal urate excretion and therefore decreased SUA levels.

In this study, we examined *Apoe* KO mice as a suitable model of ε2 homozygous subjects. We showed, for the first time, that *Apoe* KO mice had higher SUA levels than did WT mice (Fig. 1). As a plausible explanation of this phenotype, we found that *Apoe* KO mice exhibited lower renal urate excretion than WT mice, which was accompanied by little difference in intestinal urate excretion. Given that spontaneously developed hypercholesterolemia in *Apoe*-deficient mice promoted early renal dysfunction [45], the higher SUA levels and lower renal urate excretion in *Apoe* KO mice observed in this study might be associated with hypercholesterolemia-related renal dysfunction. This should be addressed in more detail in future. Also, while hyperuricemia is commonly associated with metabolic syndrome, dyslipidemia, and chronic kidney disease in humans, further studies are needed to elucidate any latent mechanisms linking the ε2 haplotype and higher SUA levels in humans.

Additionally, our multiple linear regression analyses adjusted for age, BMI, serum creatinine levels, and non-HDL-C levels revealed the ε2 haplotype to be independently associated with increased SUA levels (Table 4). Hence, despite the limited data currently available, the human phenotype in SUA levels associated with ε2 haplotype may not be explained by renal dysfunction alone. To address this point, further human studies will be required in addition to biochemical and histological investigations of the kidney using *Apoe* KO mice and/or such mice with the human *APOE* ε2.

In conclusion, we have demonstrated that the ε2 haplotype, not the ε4 haplotype, of *APOE* is associated with higher SUA levels in humans. Results of in vivo experiments suggest that renal underexcretion of urate might be involved in the observed Apoe deficiency-related SUA increase; however, further studies are required to uncover the details of the mechanisms in question. Our findings will enhance our understanding of the genetic factors affecting SUA levels in humans.
